# Clinical Cases

**Published:** 1883-11

**Authors:** E. Carleton

**Affiliations:** Honorary Member


### CLINICAL BUREAU.

### CLINICAL CASES.

*Read before the Central New York Homœopathic Medical Society, September 20th, 1883.*

E. Carleton, M. D., Honorary Member.

I. *Diabetes cured by Iodine.*—Mr. J. A. B., mid-aged, photographer, complained to me, March 1st, 1883, of passing much light-colored urine, accompanied with insatiable thirst for cold water, which he said had existed a number of months. Obtaining a specimen of his urine, I immediately submitted it to the microscochemist, Doctor Walter Y. Cowl, for examination and written statement. He found sugar. I then questioned the patient carefully, and elicited the following symptoms: Canine hunger (no emaciation yet); constipation with ineffectual urging, better from drinking milk; stools hard, knotty, dark-colored. All these, with the thirst and urinary symptoms, belong to *Iodine.* Its pathogenetic record does not include the production of sugar in the urine, and I doubt if provings of the drug have ever brought about such a result; neither have I ever heard of its being given for diabetes; but I determined to follow the indications. Hahnemann's teachings as to the superiority of the subjective symptoms, in comparison with the objective symptoms, in their importance to the homœopathic physician when selecting the similar remedy, suit me. Those who insist upon following the opposite course may do so if they choose. Then let us compare *results.*

Accordingly (March 2d), I moistened a dram of No. 25 pellets with the two hundredth potency of *Iodine,* and directed that five pellets be taken every three hours until some one or more of the symptoms should begin to improve, and then to stop taking medicine.

His bill of fare was as follows: Beef, mutton, poultry, scaly fish, oysters, clams, cheese, eggs, milk, salads, water-cresses, spinach, asparagus, cabbage, oyster-plant, radishes, beets, butter, fruits, nuts, diabetic bread; one goblet of water in twenty-four hours.

March 9th.—He reported improvement of all symptoms and medicine stopped. I advised him to stick to his diet list during the rest of the month, which he did and then said he felt well.

April 6th.—Dr. Cowl examined the urine, and stated in writing that he could find no sugar. All restrictions upon the patient were removed. He has remained well since.

II. *Hay-fever cured by Badiaga.*—Mr. W. O. A., mid-aged, bank-teller, has hay-fever annually, beginning August 18th to 20th and lasting one month. This year it first appeared on the 20th, with the usual severity, nearly disabling him for business.

Symptoms: First, sneezing and watery discharge from eyes and nose, profuse, worse on left side, alternating with stuffed-up feeling without discharge (this modality existing all through the course of the sickness); two days later, intolerable itching of eyes and nostrils; indescribable bad feeling in forehead ("*want to pull it out*"), spasmodic cough.

The patient had applied to another physician at the outset of the attack, who treated him without success for ten days. It was on the 30th day of August when he called at my office for a prescription.

In Hering's *Guiding Symptoms* (not mentioned by any other author, I believe), under *Badiaga* (*spongilla fluviatilis—fresh-water sponge*—a Russian remedy), I find the following symptoms recorded:

"7 NOSE II Sneezing, fluent coryza, stoppage at times.
I Sneezing with coryza, fluent, or thick and yellow; worst left nostril. Θ Pertussis.
Coryza and cough.
Itching of left wing of nose." *

It will be observed that the similarity between the symptoms complained of by my patient and those produced by *Badiaga* is striking.

I put a few pellets of *Badiaga*, one hundred thousandth potency (Fincke), on the patient's tongue, and gave plenty of sugar to follow.

A few days later, he called in great glee to tell me that within twelve hours after I put the dose upon his tongue, he noticed great improvement, which increased rapidly, so that he already felt nearly well. He lost no time from business. He did not need or receive a second dose of medicine. The trouble has not returned.

---

* II Means more frequently confirmed.
I " symptoms verified by cures.
Θ "The Greek letter 'theat' stands between the cured symptoms and the pathological condition, or the physiological general state, f. i., pregnancy or climacteric years. This by no means excludes the characteristic nature of the symptom in other forms of disease."—HERING.

